# Effect of Biofloc Culture on the Daily Rhythmicity of the Activity and Expression of Digestive Enzymes in Tilapia, *Oreochromis niloticus*

**DOI:** 10.1155/anu/6617425

**Published:** 2025-01-23

**Authors:** María del Carmen Monroy-Dosta, Daniel Becerril-Cortés, Juan Pablo Lazo, Arturo Mena-López, Pilar Negrete-Redondo, Eliasid Nogueda-Torres, Carmen Navarro-Guillén, José Antonio Mata-Sotres

**Affiliations:** ^1^Departamento El Hombre y su Ambiente, Universidad Autónoma Metropolitana-Unidad Xochimilco, Calzada del Hueso # 1100, Ciudad de México 04960, Mexico; ^2^Centro de Investigación Científica y de Educación Superior de Ensenada (CICESE), Carretera Ensenada-Tijuana # 3918, Zona Playitas, Ensenada, Baja California 22860, Mexico; ^3^Instituto de Ciencias Marinas de Andalucía (ICMAN-CSIC), Puerto Real, Spain

**Keywords:** biofloc, circadian rhythms, digestive enzymes, gene expression, tilapia

## Abstract

Biofloc technology (BFT) has recently attracted great attention due to minimal water exchange and reduced feed intake. This study aimed to recognize daily changes in the digestive physiology of *Oreochromis niloticus* between a traditional system and BFT. The enzyme activity of trypsin (try), chymotrypsin (chy), leucine aminopeptidase (lap), alkaline proteases (alk), lipase (lip), and amylase (amy), along with the gene expression of trypsin (*try*), chymotrypsin (*chy*), pepsin (*pep*), amylase (*amy*), and phospholipase (*pla*) were measured throughout a daily cycle. Samples were taken every 4 h in a 24 h cycle under a 12:12 L:D photoperiod. During 60 days, fish were feed three times a day (zeitgeber time, ZT: 0, 4, and 8) with a fishmeal-based diet containing 32% of crude protein and 5% of lipid, where molasses was added as a carbon source in BFT. No significant differences were found in fish performance among treatments at the end of the experiment. The activity of all tested enzymes significantly (*p* < 0.05) increases during the dark period in both treatments, where the same activity pattern was found in try and lip. The maximum expression levels of digestive gene enzymes between treatments show a marked effect dependent on the presence of light and dark phases. The cosinor analysis showed an activity in try, lap, and lip with a significant rhythmicity (*p* < 0.05). Our results demonstrate that some processes related to the digestive physiology of tilapia that respond directly to daily rhythmicity are modified under the constant presence of feed in BFT. These findings should be considered when establishing new optimized culture protocols.

**Summary**

• Tilapia exhibited an increase in enzymatic activity during the dark phase in biofloc.
• Enzymatic activity and expression of the digestive enzymes showed rhythmic patterns.
• One of the first studies to analyze digestive rhythmicity in biofloc culture.

## 1. Introduction

Throughout evolution, organisms have developed mechanisms capable of generating biological rhythms, bringing the possibility of adapting their physiology to anticipate environmental 24-h cyclical changes [1]. These cycles, or circadian rhythms, are physical, physiological, and behavioral changes following a 24-h cycle with important effects on movement, metabolism, reproduction, immune response, and other physiological aspects [2]. Photoperiod (direct influence of light and darkness cycle) and thermal oscillations are key environmental variables driving circadian cycles. In the case of teleost fishes, the functional organization of this circadian system is based on the presence of physiological oscillators or “clocks,” with a common molecular basis throughout the phylogeny and resides in highly conserved rhythms of gene expression, which form feedback loops with a periodicity close to 24 h [3, 4].

In relation to fish feeding activity, the knowledge of the phase of the day in which the animals prefer to feed has an important productive implication, especially if we consider that fish do not show a sustained feeding activity during the complete day but concentrate their feeding activity in a specific phase, presenting diurnal, nocturnal, or dual patterns. The digestive system is prepared to anticipate this event, with the production of digestive enzymes activated before starting the feeding process [5–11]. To make the most of nutrient acquisition, some fishes showed an anticipatory activity and feeding patterns, as showed in the Atlantic salmon (*Salmo salar*), goldfish (*Carassius auratus*), and European seabass (*Dicentrarchus labrax*) [5, 12, 13]. Hence, the appropriate knowledge of feeding behavior provides essential information for the correct design of new feeding protocols for each of the species [14, 15]. Likewise, various studies pointed out that under culture conditions, although providing a continuous supply of food, fish continue to show feeding rhythms [16, 17].

Looking for a more sustainable aquaculture, new culture systems have emerged, such as the so-called biofloc technology (BFT) [18]. This recent technology promotes the production of microbial flocs that utilize nitrogenous and feed waste from fish and an external carbon source to produce microbial protein as a natural food available 24 h a day for fish, with a minimal or no water turnover [19–21]. Additionally, BFT provides nutrients such as amino acids, saturated and unsaturated fatty acids, as well as vitamins and minerals to the culture [22]. Many questions arise about whether fish feeding rhythms, with their concomitant activity and expression of digestive enzyme rhythms, are maintained or modified under this type of culture system.

The Nile tilapia (*Oreochromis niloticus*) is one of the three most widely farmed fish species in the world due to their ability to adapt to a wide range of environmental conditions, as well as to different farming systems (such as BFT), which has led to their cultivation in more than 80 countries [23, 24]. It offers meat of excellent nutritional quality, good flavor, firm texture, and little bone. The price stability and marketability have allowed production to quadruple in the last 10 years [24–26].

Hence, the aim of this study was to determine whether juveniles of *O. niloticus* cultured in biofloc modify their daily rhythms of production of digestive enzymes, which will allow for optimizing current culture protocols.

## 2. Materials and Methods

### 2.1. Rearing Conditions

In total, 150 juvenile Nile tilapias weighing 1.05 ± 0.13 g were obtained from the Zacatepec Aquaculture Center (Morelos, México) and transported to the facilities of the Laboratory of Chemical Analysis of Live Food of the Universidad Autónoma Metropolitana, Xochimilco. Fish were gradually adapted to the experimental conditions during 15 days, based on the temperature (24.1 ± 0.8°C) and dissolved oxygen (DO) (6.7 ± 0.4 mg L^−1^) of the hatchery. Photoperiod followed a 12 L:12 D illumination regime, light was switched on at zeitgeber time (ZT) 0 and off at ZT 12 (8:00 and 20:00 h local time, respectively). Fish were feed three times a day (ZT 0, 4, and 8) to apparent satiation with a fishmeal-based diet containing 32% of crude protein and 5% of lipids (Alimentos del Pedregal, Toluca, Estado de México, México) [27].

After the acclimatization period, the fish were randomly distributed in 6 rectangular tanks of 100 L capacity (15 fish/tank) under the aforementioned environmental conditions. Two treatments were considered during a 60-day trial: control (traditional clear water culture) and BFT. The organisms under experimental conditions were fed at a feeding rate of 10% body weight, adjusted every 15 days in relation to the growth of the organisms. Experimental treatments were randomly assigned to three replicate tanks.

Daily, DO, temperature, and pH were monitored at ZT 2 (Hanna, model HI 9829). Nitrites, nitrates, and total ammonia nitrogen (TAN) were measured every 10 days (Hanna, model HI83203). Water quality parameters in both treatments were maintained within suitable ranges for this species. Water replacement was carried out every third day at a renewal rate of 40% for the control group. At the end of the experiment, the control treatment showed an average temperature: 29.4 ± 0.16°C, DO: 4.75 ± 0.04 mg L^−1^, TAN: 0.71 ± 0.04 mg L^−1^, nitrite: 0.84 ± 0.01 mg L^−1^, nitrate: 21.5 ± 0.29 mg L^−1^, and pH = 7.04 ± 0.05 (mean ± SE).

In the biofloc treatments, the external carbon source was added daily as molasses in a 20:1 C/N ratio to guarantee floc formation, following the system requirement calculations recommended [28]. And adjusting the addition of molasses every 15 days according to the increase in food rations in relation to the biomass of the tank. No water replacement was performed; only evaporation loss was compensated.

### 2.2. Fish Sampling and Response Variables

Daily cycles in the gastrointestinal tract were examined to determine the relation between the activity of digestive enzymes and gene expression. On the sampling day, three fish per tank (*n* = 9) were sampled every 4 h until completing a 24 h cycle, starting at ZT 0. Fish were fed 15 min after sampling at ZT 0, 4, and 8 [29]. Fish were euthanized using a two-step procedure, first quickly chilled on ice until unconsciousness with a subsequent cut in the spinal column to the side of the skull [30]. Fish were individually measured (mm, standard length, SL) and weighted (g). The organism's liver was weighed individually. Afterward, the digestive tract (stomach and intestine) per fish was placed on a cold plate, weighed, and stored separately at −80°C until analysis.

### 2.3. Enzyme Activity Analyses

The procedures to obtain for measuring fish enzymatic activities are fully described in Fuentes-Quesada et al. [31] and Fuentes-Quesada and Lazo [32]. In summary, 1 mM of BAPNA (Sigma, B-4875) was used as a substrate for measure trypsin (try) activity following Erlanger, Kokowsky, and Cohen [33]. The activity of chymotrypsin (chy) was determined using 0.56 mM of BTEE (Sigma, 13110-F) adapted from Applebaum et al. [34]. While for the activity of the leucine aminopeptidase (lap) was measured using 1.2 mM of reactive grade lap (Sigma, L-9125) [35]. The activity of lipase (lip) was determined using 0.56 mM of myristate (4-nitrophenyl, Sigma, 70124) [28]. The activity of amylase (amy) was determined by using 1% starch (Sigma, S9765) (WBC, 1993). Using 2% of azocasein (Sigma, A2765), total alkaline proteases (alk) activity was measured [36]. To determine the concentration of soluble protein, the Bradford method was used (BIO-RAD, Protein assay). All the tests per sample were conducted in triplicate.

All digestive enzyme reactions were determined kinetically in a microplate reader (Varioskan Flash, Thermo Scientific) and analyzed using the SkanIt software (RE 2.4.5). In all cases, a distilled water sample was used as a blank, and a positive control was used with commercial enzymes at 1 mg mL^−1^. All enzymatic activities are expressed in enzyme units (U), where one unit is the increase of 0.1 units of absorbance min^−1^ [37].

### 2.4. Gene Expression Quantification

The protocols to obtain molecular RNA extracts from the liver and for determine the expression levels are detailed in Mata-Sotres et al. [9] and Garnica-Gómez, Mata-Sotres, and Lazo [38]. In summary, to determine the expression levels of *try*, *chy*, pepsinogen (*pep*), *amy*, and phospholipase (*pla*), six hepatic samples per measured ZT-time and were kept in RNAlater (Ambion) and stored at −80°C. Total RNA was extracted individually by the organism, using the NucleoSpin RNA kit following manufacturer procedures (Macherey-Nagel). The quality and quantity of RNA were determined spectrophotometrically using a Nanodrop LITE (Thermo Fisher Scientific INC., Wilmington, USA), where only the samples with an OD260nm–OD280nm ratios between 1.90 and 2.10 were used for subsequent processes.

The high-capacity cDNA reverse transcription kit (Applied Biosystems; Carlsbad, CA, USA) was used for the reverse transcription of 500 ng total RNA in a 20 μL reaction, following manufacturers' procedures. The qPCR reactions were performed with 1 ng of cDNA, 200 nM of sense and antisense primers (200 nM, Table 1), and a green fluorescent dye (SYBR, Applied Biosystems).

The *ΔΔ*CT method [39] was used to calculate relative gene expression based on the determination of the CT values, as described by Mata-Sotres et al. [29] and Araújo et al. [40], using StepOnePlus System (96-well reaction plates Applied Biosystems).

A cDNA of control treatment was used as a calibrator in all reactions, including the reference gene, as reported by Becerril-Cortes et al. [27] and Garnica-Gómez, Mata-Sotres, and Lazo [38]. GenBank accession numbers for the studied genes are AY510093.1 for *try*, XM_019359917.1 for *chy*, XM_003442057.5 for *pla*, AJ555243.2 for *amy*, JQ043215.1 for *pep*. and KJ126772.1 for the internal reference gene *β*-actin (*actb*). Primers were designed using Primer3 Software version 0.4.0. Table 1 shows the primer sequences and product sizes.

### 2.5. Data Analysis

The statistical assumptions of normality and homogeneity were calculated using Shapiro–Wilks and Barlett tests, respectively [41], followed by one-way ANOVA and a Tukey rank test in growth performance, digestive enzymatic activity, and gene expression levels. A significance level of *p*  < 0.05 was used in all cases (STATISTICA 8.0, StatSoft, Inc. USA). All results are reported as mean ± standard error of the mean (SEM).

Cosinor analysis was used to determine the rhythmicity (El Temps, Dr. Diez-Noguera, Barcelona), where a significant rhythmicity is assumed when sinus-fit and amplitude are differing from 0 in the cosine, mesor, amplitude, and acrophase.

## 3. Results

At the end of the experiment, the control treatment showed an average temperature: 29.4 ± 0.16°C, DO: 6.7 ± 0.04 mg L^−1^, TAN: 0.71 ± 0.04 mg L^−1^, nitrite: 0.84 ± 0.01 mg L^−1^, nitrate: 21.5 ± 0.29 mg L^−1^, and pH = 7.04 ± 0.05 (mean ± SE). Whereas for BFT culture conditions were temperature: 29.7 ± 0.16°C, DO: 5.71 ± 0.05 mg L^−1^, TAN: 0.52 ± 0.04 mg L^−1^, nitrite: 0.89 ± 0.04 mg L^−1^, nitrate: 13.37 ± 0.21 mg L^−1^, and pH = 7.55 ± 0.07 (mean ± SE).

### 3.1. Fish Performance

At the end of the experimental period, no clear effects on fish growth related to the culture method were found. Both treatments showed the same increase in biomass, with no statistical difference between treatments in FI (*p*=0.39), SGR (*p*=0.94), FCR (*p*=0.58), CF (*p*=0.83), and TGC (*p*=0.82) (Table 2). Similarly, weight gain was obtained for control (125.65 ± 10.26 g) and BFT (117.54 ± 11.90 g) treatments. Although HSI and VSI showed a slight increase in fish from BFT, no significant differences were observed (*p*=0.16 and 0.18, respectively). Likewise, survival was not affected by the culture system (Table 2).

### 3.2. Digestive Enzymes Activity

**Figure 1:**
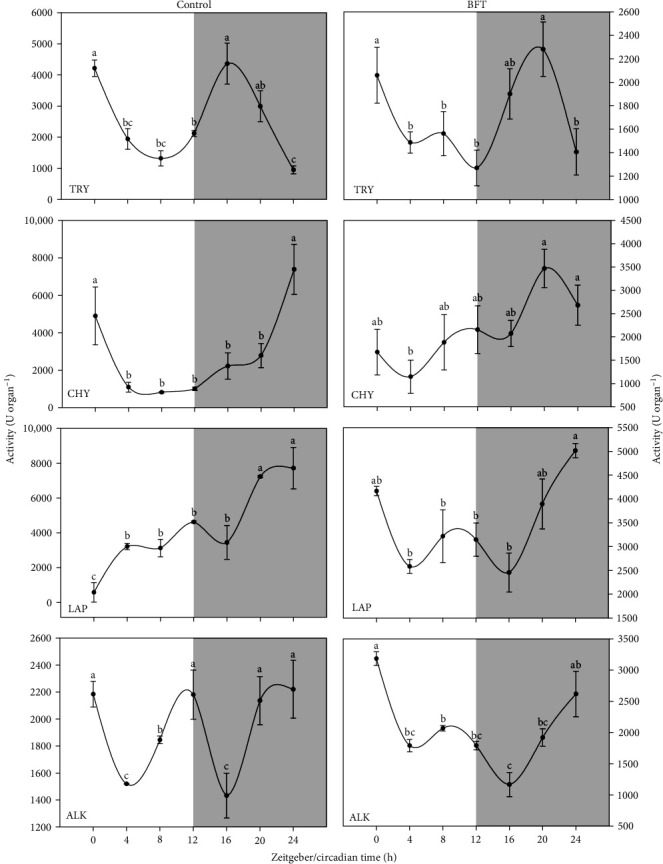
Proteases activity (U organ^−1^) for trypsin (try), chymotrypsin (chy), L-aminopeptidase (lap), and total alkaline (alk) proteases in *O. niloticus*. Different letters represent significantly different values (*p* < 0.05) within the same organ (*n* = 6, mean ± SEM). The white and gray part of the graph symbolizes the light and dark periods of the cycle. SEM, standard error of the mean.

**Figure 2:**
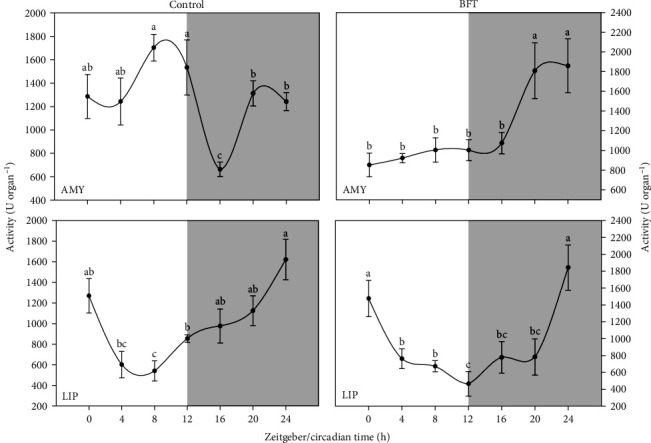
Total amylase (amy) and lipase (lip) activity (U organ^−1^) in *O. niloticus*. Different letters represent significantly different values (*p* < 0.05) within the same organ (*n* = 6, mean ± SEM). The white and gray part of the graph symbolizes the light and dark periods of the cycle. SEM, standard error of the mean.

The activity levels of all studied digestive enzymes were significantly affected by photoperiod in both treatments (Figures 1 and 2). Specifically, try activity shown the most similar activity pattern between treatments, with a marked reduction as the light hours of the photoperiod progress and a subsequent significant increase (*p* < 0.05) in the dark phase, reaching the maximum activity level at ZT 16 and ZT 20 for control and BFT, respectively. Although there were no similarities in the activity patterns between control and BFT in the rest of the proteases, a significant increase in activity near the end of the dark phase (ZT 20 and ZT 24) was observed for chy, lap, and alk (Figure 1).

In the case of lip, the same activity pattern was observed for both treatments, with a trend to decrease along the day, showing significantly lower values near the end of the lighting phase (ZT 8 and ZT 12 for control and BFT, respectively), with a subsequent significant increase towards the end of the daily cycle, showing a marked “U” shape throughout the cycle (Figure 2). While for amy, a significant increase was observed at the end of the dark (ZT 20 and ZT 24) for both treatments.

### 3.3. Gene Expression

**Figure 3:**
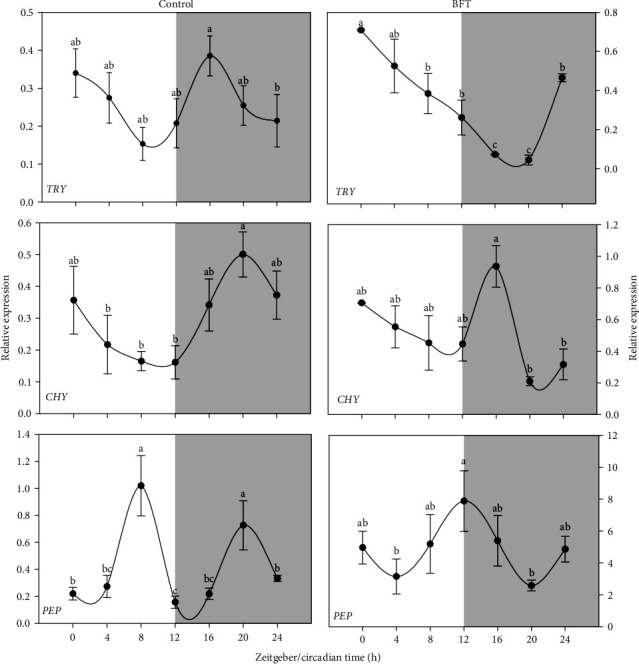
Proteases relative expression pattern of trypsin (*try*), chymotrypsin (*chy*), and pepsinogen (*pep*) in *O. niloticus*. Different superscript letters represent significantly different values (*p* < 0.05) (*n* = 6, mean ± SEM). The white and gray part of the graph symbolizes the light and dark periods of the cycle. SEM, standard error of the mean.

The expression patterns for the analyzed proteases are represented in Figure 3. In the case of alk proteases, no similar expression patterns were found in *try* and *chy* for control or BFT treatments. Nevertheless, in the case of *chy*, a peak of expression (*p* < 0.05) was observed close to the middle of the dark period, at ZT 20 for control and ZT16 for BFT. Whereas in the acid protease expression, *pep* showed a bimodal peak of just in the middle of the light and darkness periods at ZT 8 and ZT 20 for control treatment, contrasting with pep expression in the BFT group where a single peak was observed just at the moment of the light transition at ZT 12 (Figure 3).

**Figure 4:**
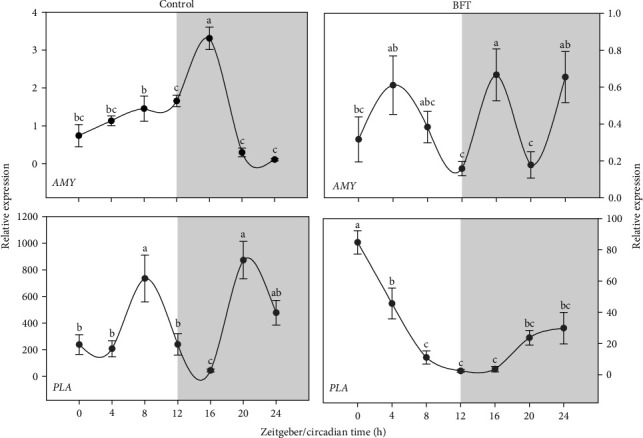
Amylases (*amy*) and phospholipase (*pla*) relative expression in *O. niloticus*. Different superscript letters represent significantly different values (*p* < 0.05) (*n* = 6, mean ± SEM). The white and gray part of the graph symbolizes the light and dark periods of the cycle. SEM, standard error of the mean.

In the case of *amy* expression, a peak of relative gene expression was observed for the control group at ZT 16, followed by a marked significant decrease (*p* < 0.05) along the rest of the dark phase, contrasting with the expression pulses found in BFT with a series of expression peaks (approximately every 8 h) (Figure 4). Relative gene expression pattern of *pla* in control fish showed a bimodal peak (*p* < 0.05) close to the middle of each of the lighting phases (ZT 8 and ZT 20), contrasting with the *pla* expression pattern in BFT with a continuous significant decrease throughout the daily cycle (Figure 4).

### 3.4. Daily Rhythmicity

**Figure 5:**
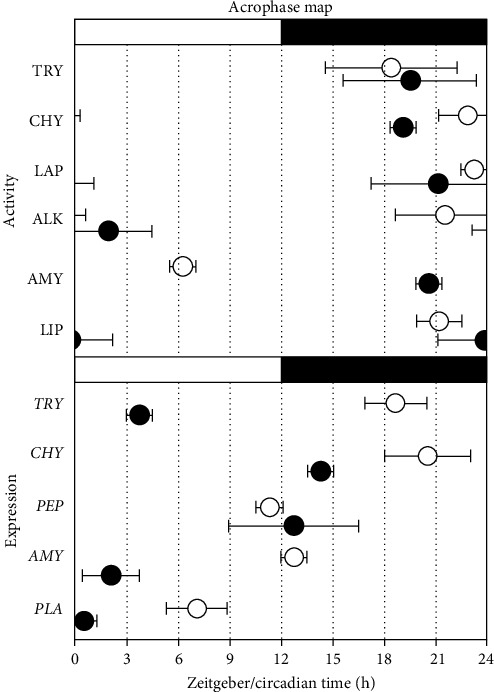
Acrophase maps of digestive enzymes activity and expression in *O. niloticus*. White and black bars on the top represent the light and dark periods under the LD regime. White dots represent control treatment, and black dots represent BFT treatment. BFT, biofloc technology.

Significant rhythmicity (*p* < 0.05) was found in the daily patterns of try, chy, and alk for the control group (Table 3). By contrast, for the BFT group, significant rhythmicity was found for try, lap, and lip. It is worth mentioning that try was the only proteolytic enzyme showing a daily rhythmicity in both treatments (Table 3), with acrophase values similar between both experimental groups. In relation to the enzymatic activity of amy, no significant daily rhythmicity was found for any of the experimental treatments. In contrast, lip activity showed a significant rhythmicity (*p* < 0.05) in the control treatment as well as in BFT (Table 3), with the acrophase located in the second half of the darkness period for both treatments (Figure 5).

Regarding gene expression rhythmicity, daily expression levels of *try*, *chy*, and *pla* were statistically significant rhythmic in control fish (*p* < 0.05). Contrasting with BFT where only the expression of *pep* and *amy* showed a significant rhythmicity (Table 3). The significant acrophases in control treatment occurred at the middle/end of the day (for *try* and *chy*) and in the middle of the light phase (for *pla*), while for BFT, acrophases occurred at the beginning of the light cycle (for *amy*) and around the transition from light to dark phases (for *pep*) (Figure 5).

## 4. Discussion

The enormous expansion of aquaculture worldwide has generated the need to develop new, more sustainable culture systems and, consequently, a better understanding of the physiological processes of fish under these new rearing technologies to guarantee an improvement in digestive efficiency impacting nutrient absorption, with benefits in survival, growth and, ultimately, in productivity [24, 42–44]. In this sense, various studies indicated that one of the best production strategies in fish aquaculture is the biofloc system, which ensures the promotion of flocs rich in microbial protein available 24 h a day, generating positive effects on digestive enzymatic activity, feed conversion, growth, immunity, oxidative stress, among others [45–47].

The results of the present study related to growth parameters did not show significant differences between the control and BFT treatments, contrasting with what reported by Khanjani et al. [48] and Khanjani and Sharifinia [49], where tilapia grown in BFT resulted in improved production parameters due to the constant presence of flocs rich in bioactive compounds such as carotenoids, chlorophylls, and phytosteroids [44, 49]. Where tilapias tend to increase growth due to the direct effect of lighting hours in the photoperiod [49]. However, when dietary protein levels are greater than 35%, it may generate stress associated with what is known as “protein overload,” which can reduce growth performance, feed intake and even affect the activity of proteases in juvenile Nile tilapia, resulting in a decrease in the final weight, weight gain and SGR as protein levels increase [50]. Therefore, the interaction between high protein levels in the formulated feed and BFT may generate nutritional stress, which suggests that a reduction in protein levels in the pellets can favor the growth of tilapia in BFT culture. The lack of differences in growth performance between feeding strategies, including the number of meals per day, self-feeding, apparent satiety, or fixed daily ration, has also been reported for the arapaima (*Arapaima gigas*) [51].

Many fish species have the ability to modify their activity rhythms from diurnal to nocturnal or vice versa if they are trained to feed at certain times within the light or dark phases [52, 53]. Based on this premise, the Nile tilapia can present diurnal, nocturnal, or mixed patterns of activity and feeding [5]. In the present investigation, tilapia juveniles under control and BFT treatments showed crepuscular patterns of digestive enzyme activity regardless of the presence of constant feed (i.e., in the BFT). It is worth mentioning that digestive enzyme activity is an indirect way of measuring the feeding rates of teleost fish since, in most cases, a direct postprandial response is presented [8, 14, 54]. Similar patterns of nocturnal enzymatic activity have been reported for tambaqui (*Colossoma macropomum*) and tilapia previously [52, 55]. Although various feeding strategies have been reported for tilapia in relation to the photoperiod [5, 51], under stable photoperiod conditions, tilapia increases its activity at dusk at temperatures close to 30°C, while at lower temperatures (close to 25°C), the activity becomes diurnal [56, 57]. In addition, under BFT conditions at 26°C, tilapia increases its ingestion rate and enzyme activity under the presence of hours of lighting [49]. Therefore, in addition to the constant presence of flocs in the water, it is recommended to feed during the final phase of lighting hours close to the beginning of the dark hours to optimize the performance of the tilapia. One aspect to consider is that feed represents a high percentage of the total production costs in tilapia [58], so feeding rhythms must be considered to optimize feeding protocols and improve production while reducing feed costs [59].

The ability of an organism to digest food particles depends on the amounts and appropriate functionality of digestive enzymes, which in turn are controlled by internal and external signals such as light/dark and feeding/fasting cycles, which typically present circadian patterns [42, 49]. While the liver responds directly to lighting and feeding time to adjust its metabolic functions, the intestine responds directly to feeding time by modifying, among other things, the length of its microvilli to optimize nutrient absorption [60]. In this study, the levels of protease activity resulted in a progressive increase during the dark period for try, chy, lap, and alk in both treatments, peaking at the beginning of the light period (ZT 0) and/or near the middle of the dark period. Similar patterns have been reported by Montoya et al. [6], who observed in the Nile tilapia greater activity of proteases (i.e., try and chy) during the night, with a decrease in activity in the first hours of the morning and subsequently increase their activity just before starting the feeding processes, as observed in the present study. Likewise, the secretion and activity of proteases, lipases and amylases in organisms undergo changes in response to the amount of food offered and in relation to the feeding technique used. In the present study, under BFT, the fish were able to feed to satiety, where the digestive enzyme activity patterns were modified in relation to the control, which demonstrates the plasticity of the circadian system and its synchronizers in tilapia [7, 49, 61–64].

In the present research, the activity pattern of specific digestive enzymes resulted in variations in relation to the culture system. For example, in the case of the control treatment, a rhythmicity was observed for try, chy, alk, and lip, while for BFT fish rhythmic oscillations were observed for try, lap, and lip. Because enzymes such as chy and try respond directly to the protein levels present in the gut [49, 52, 64], the rhythmic activity pattern observed for try, suggests that there is a regulation of daily feeding activity despite having the constant presence of food in the BFT treatment. This variation in rhythmicity under constant feeding conditions has been previously observed in tambaqui, which, based on its enzymatic activity rhythms, suggests a nighttime feeding activity [50]. Furthermore, de Oliveira et al. [64] reported similar activity patterns of acid protease, try, and chy, when tilapia was fed at similar times than the present study during the lighting phase. Furthermore, similar patterns of protease activity have been found in species under constant presence of food such as blue bream (*Ballerus ballerus*) or in amy for the tambaqui fed on a free demand system through the use of automatic self-feeders [65, 66]. Likewise, Hernández-Mancipe et al. [22] reported that in a BFT system, the digestive enzyme activity may increase due to exoenzymes produced by the microbial community present in the system, which might explain the differences in activity patterns found for some digestive enzymes between BFT and control treatments. However, based on the rhythmicity patterns of digestive enzyme activity found for both treatments, an effect of genetic preprogramming that responds to other endogenous factors is observed since the constant presence of food in BFT does not modify the maximum hours of activity (acrophases) in comparison to control [14, 16, 67–70].

In relation to the gene expression of *try*, *chy*, and *pla*, a significant rhythmicity was observed in their daily expression levels in the control treatment. While in the relative gene expression patterns for BFT, only *amy* and *pep* showed this significant rhythmicity. However, despite not having a significant rhythmicity for all genes, the presence of expression patterns favors the digestion of certain nutrients throughout the daily cycle for both treatments [17, 67, 71, 72].

The *try* plays an essential role in controlling the activity of other pancreatic enzymes in teleosts. The presence of protein in the tract promotes and increase in *try* activity in the intestine and the subsequent activation of other digestive zymogens, being an essential process for protein digestion [50, 73]. Furthermore, it has been reported that at protein levels higher than 35%, tilapia juveniles reduce *try* expression levels and concomitantly increasing *chy* expression [48], which can generate the modification in the daily patterns of both proteases. Furthermore, the difference found between daily patterns of activity and expression of digestive enzymes suggests the intervention of posttranscriptional/posttranslational mechanisms [74]. This disagreement between expression and activity has been previously reported for several fish species, such as the yellowhead catfish (*Pelteobagrus fulvidraco*), gilthead sea bream (*Sparus aurata*), and the white seabream (*Diplodus sargus*) [14, 54, 74, 75]. Relative gene expression patterns of *pep* in the BFT treatment are similar to those reported for gilthead sea bream when fed constantly during the lighting phase, where the constant presence of food resulted in longer gastric digestion times [76]. Taken together, these results show that tilapia has the ability to posttranscriptionally modify expression patterns to optimize digestive functionality, as previously reported for gilthead sea bream [74].

In conclusion, *O. niloticus* exhibits daily patterns in activity and expression of the main digestive enzymes that were modified by the constant presence of food in the BFT system, having nocturnal digestive enzyme activity for both treatments. The above results can be considered when establishing new cultivation and feeding protocols for this species under BFT conditions.

## Figures and Tables

**Table 1:** Primers pairs used for q-PCR primer sequences, amplicon sizes in base pairs (bp), reaction efficiencies (*E*), and Pearson's coefficients of determination (*R*^2^) are indicated.

Gene (symbol)	Fwd sequence (5′−3′)	Rv sequence (5′–3′)	Size (bp)	*E*	*R* ^2^
*bact*	GAG CGT GGC TAC TCC TTC AC	GCA GGA TTC CAT ACC AAG GA	234	0.98	0.99
*try*	ATA TGC TGC TCC CAT TGA GG	CTG GTA GCA GTG AGC AGC AG	157	0.95	0.99
*chy*	GTG ATT GCT GGA GAG CAC AA	TGT CAG TAG TGT CGG CAA GG	193	0.91	0.99
*pep*	CTT GTG GGT CCC TTC AGT GT	GCT TCT GTC TGG CTC AAT CC	213	0.95	0.99
*amy*	TCA ACC ACG TGT GTG GAT CT	CAA TCT CAC CCA CTC CCA GT	151	1.05	0.94
*pla*	GTT GGC AGG AGC AAT CAA AT	CAA GAC GTT CTG CAT CTC CA	158	0.94	0.98

**Table 2:** Growth performance of *O. niloticus* under different culture protocols.

	Control	BFT	PSE	*p*-Value
Initial weight (g)	1.52 ± 0.20	1.71 ± 0.13	0.11	0.47
Final weight (g)	3.43 ± 0.37	3.72 ± 0.01	0.17	0.55
Weight gain (%)	125.65 ± 10.26	117.54 ± 11.90	7.03	0.95
^a^FI (% day^−1^)	2.17 ± 0.39	1.80 ± 0.06	0.19	0.39
^b^SGR (% day^−1^)	1.52 ± 0.21	1.50 ± 0.24	0.15	0.94
^c^FCR	1.07 ± 0.03	0.93 ± 0.19	0.38	0.58
^d^CF	3.22 ± 0.14	3.27 ± 0.17	0.10	0.83
^e^TGC	0.36 ± 0.08	0.38 ± 0.05	0.04	0.82
^f^HSI%	15.90 ± 0.32	17.34 ± 0.80	0.50	0.16
^g^VSI%	16.79 ± 0.61	18.08 ± 0.53	0.46	0.18
Survival (%)	96.67 ± 1.67	97.78 ± 2.22	1.27	0.74

*Note:* Values are presented as means ± SE. Also, pooled standard error (PSE) is given from three replicates per treatment and *p* values resulting from a one-way ANOVA test are also provided. Different superscript letters represent significantly different values (*p*  < 0.05) within the same row.

^a^Feed Intake = (FI, % day^−1^) = 100 × (total amount of the feed consumed/((initial body weight + final body weight)/2)/days).

^b^Specific growth rate (SGR, % day^−1^) = 100 × ((ln final weight − ln initial weight) × number of days).

^c^Feed conversion ratio (FCR) = total feed consumed/wet weight gained.

^d^Condition factor (CF) = (final body weight/body length^3^) × 100.

^e^Thermal growth coefficient (TGC) = [(final weight ⅓ − initial weight ⅓)/(*T* °C days)] × 1000.

^f^Hepatosomatic index (HSI, %) = (hepatopancreas weight/body weight) × 100.

^g^Viscerosomatic index (VSI, %) = (viscera weight/body weight) × 100.

**Table 3:** Acrophase (ACR), mesor (MES), and amplitude (AMP) (mean = 95% confidence interval) based on cosinor analysis for feed intake, enzyme activity, and gene expression of the main digestive enzymes in *O. niloticus*.

Activity	ACR	MES	AMP	*p*-Value
Control				
try	18.10 ± 4.41	2553.15 ± 577.58	1181.72 ± 1099.22	0.03
chy	22.57 ± 3.01	2564.26 ± 931.60	2452.41 ± 1598.12	<0.01
lap	23.21 ± —	3387.51 ± 406.94	779.08 ± —	0.24
alk	22.30 ± 3.04	1913.41 ± 181.16	137.86 ± 41.30	<0.01
amy	6.67 ± —	1290.88 ± 160.94	284.58 ± —	0.69
lip	21.12 ± 2.85	956.34 ± 144.97	405.39 ± 260.34	<0.01
BFT				
try	19.79 ± 4.86	1686.14 ± 184.85	366.86 ± 344.97	0.03
chy	18.50 ± —	2140.7 ± 406.44	731.22 ± —	0.06
lap	21.35 ± 4.28	4807.00 ± 737.11	1957.94 ± 1314.40	<0.01
alk	1.43 ± 2.88	1995.65 ± 241.17	605.20 ± —	0.59
amy	20.49 ± —	1187.91 ± 201.00	348.19 ± —	0.06
lip	23.72 ± 2.87	897.07 ± 189.17	502.41 ± 317.82	<0.01

**Expression**	**ACR**	**MES**	**AMP**	***p*-Value**

Control				
* try*	19.25 ± 1.90	0.25 ± 0.05	0.64 ± 0.14	<0.01
* chy*	20.39 ± 2.70	0.28 ± 0.05	0.16 ± 0.10	<0.01
* pep*	11.70 ± —	0.42 ± 0.16	0.10 ± —	0.67
* amy*	13.13 ± —	1.38 ± 0.37	1.01 ± —	0.64
* pla*	7.66 ± 1.90	0.32 ± 0.07	0.27 ± 0.14	<0.01
BFT				
* try*	3.66 ± —	0.32 ± 0.07	0.27 ± —	0.26
* chy*	14.09 ± —	0.52 ± 0.13	0.70 ± —	0.68
* pep*	13.13 ± 4.41	5.06 ± 1.13	0.10 ± 0.80	0.04
* amy*	2.17 ± 1.66	0.41 ± 0.12	0.37 ± 0.07	<0.01
* pla*	0.47 ± —	394.25 ± 158.91	58.84 ± —	0.84

*Note:* Mesor and amplitude are given as activity or relative expression values, and acrophases as zeitgeber/circadian time (ZT/CT) hours. Trypsin (try), chymotrypsin (chy), aminopeptidase (lap), alkaline (alk) proteases, amylases (amy), lipases (lips), pepsinogen (pep), and phospholipase (pla).

## Data Availability

Data will be made available upon request.
